# Endotracheal Intubation Among the Critically Ill: Protocol for a Multicenter, Observational, Prospective Study

**DOI:** 10.2196/11101

**Published:** 2018-12-07

**Authors:** Nathan Smischney, Rahul Kashyap, Mohamed Seisa, Darrell Schroeder, Daniel Diedrich

**Affiliations:** 1 Department of Anesthesiology, Mayo Clinic Rochester, MN United States

**Keywords:** airway, endotracheal intubation, hemodynamics, intensive care unit, multi-center, prospective study

## Abstract

**Background:**

Endotracheal intubation can occur in up to 60% of critically ill patients. Despite the frequency with which endotracheal intubation occurs, the current practice is largely unknown. This is relevant, as advances in airway equipment (ie, video laryngoscopes) have become more prevalent, leading to possible improvement of care delivered during this process. In addition to new devices, a greater emphasis on airway plans and choices in sedation have evolved, although the influence on patient morbidity and mortality is largely unknown.

**Objective:**

This study aims to derive and validate prediction models for immediate airway and hemodynamic complications of intensive care unit intubations.

**Methods:**

A multicenter, observational, prospective study of adult critically ill patients admitted to both medical and surgical intensive care units (ICUs) was conducted. Participating ICU sites were located throughout eight health and human services regions of the United States for which endotracheal intubation was needed. A steering committee composed of both anesthesia and pulmonary critical care physicians proposed a core set of data variables. These variables were incorporated into a data collection form to be used within the multiple, participating ICUs across the United States during the time of intubation. The data collection form consisted of two basic components, focusing on airway management and hemodynamic management. The form was generated using RedCap and distributed to the participating centers. Quality checks on the dataset were performed several times with each center, such that they arrived at less than 10% missing values for each data variable; the checks were subsequently entered into a database.

**Results:**

The study is currently undergoing data analysis. Results are expected in November 2018 with publication to follow thereafter. The study protocol has not yet undergone peer review by a funding body.

**Conclusions:**

The overall goal of this multicenter prospective study is to develop a scoring system for peri-intubation, hemodynamic, and airway-related complications so we can stratify those patients at greatest risk for decompensation as a result of these complications. This will allow critical care physicians to be better prepared in addressing these occurrences and will allow them to improve the quality of care delivered to the critically ill.

**Trial Registration:**

ClinicalTrials.gov NCT02508948; https://clinicaltrials.gov/ct2/show/NCT02508948 (Archived by WebCite at http://www.webcitation.org/73Oj6cTFu)

**International Registered Report Identifier (IRRID):**

RR1-10.2196/11101

## Introduction

### Significance

When compared to other settings, such as the operating room, endotracheal intubation in the intensive care unit (ICU) carries with it a higher morbidity and mortality, likely due to many factors, including a lack of physiologic reserve [1-3]. For example, the incidence of a difficult airway in the ICU may be as high as 23% [2]. Unwanted effects associated with endotracheal intubations performed in the ICU include, but are not limited to, arterial desaturation, cardiovascular decompensation, esophageal intubation, regurgitation of gastric contents, and cardiac arrest [3-5]. Additionally, as intubation attempts increase, the rate of complications also increases [6]. In recognition of the above, institutions across the country have developed intubation bundles to reduce these unwanted effects. Moreover, the use of a systematic approach to, or protocol for, endotracheal intubation may reduce intubation complications [7-9]. This was recently demonstrated in a trial utilizing an intubation protocol, whereby immediate life-threatening complications surrounding ICU intubations were reduced [10].

### Challenges in Endotracheal Intubation

Recently, there have been a variety of new devices emerging that are designed to assist with a difficult airway, such as video laryngoscopes. These devices have been reported to reduce unwanted effects of endotracheal intubation (ie, a failed airway). In addition to new devices, intubation checklists and sedative choices have undergone changes with uncertain effects on patient morbidity and mortality. Currently, many clinicians use the newer devices, such as video laryngoscopes, as evidence indicates that these devices result in better laryngeal view and improved intubation difficulty score with lower risk of a failed airway as compared to conventional techniques (ie, direct laryngoscopy) [11-15]. Moreover, these newer devices are user friendly even in unfamiliar hands [16]. A recent meta-analysis comparing video laryngoscopy with direct laryngoscopy reported similar findings, where video laryngoscopy reduced the risk of difficult airway, Cormack 3/4 grades, and esophageal intubation, but increased the first-attempt success rate. Additional outcomes, such as severe hypoxemia, severe cardiovascular collapse, or airway injury, were not different between the two techniques [17]. Moreover, video laryngoscopy maintains its effectiveness when used during an emergency [18]. Despite the evidence of positive outcomes for the newer devices, not all providers utilize these modalities, possibly due to inexperience with the newer techniques or evidence suggesting no benefit [19,20]. As an example, a recent study surveying Canadian resuscitation physicians demonstrated that most use direct laryngoscopy as their go-to technique for emergent endotracheal intubations [21]. Similarly, ICU physicians in Israel, when surveyed, seem to prefer fiber-optic intubation for routine airway management [22].

### Medications and Procedural Advances in the Field

Along the same line as airway equipment, sedatives used during endotracheal intubation have evolved over time. Over the years, evidence has suggested that the use of etomidate in the critically ill, especially in sepsis, may be associated with increased morbidity and mortality [23-25]. However, other studies find no associations with etomidate and patient outcomes [26,27]. Etomidate traditionally has been the preferred induction drug because of its favorable hemodynamic profile. However, with mounting evidence for adrenal suppression and possible associations with mortality in septic patients, the clinician now struggles with the ideal sedative for endotracheal intubation [28-30]. Other agents and/or admixtures have shown promise [31,32]. Not only has the choice of induction medication changed in recent years, but current evidence suggests that the use of paralytics may help facilitate endotracheal intubation [33,34]. In addition, paralytics are now recommended in the setting of acute respiratory failure, with evidence demonstrating improved outcomes [35]. Thus, using paralytics in a patient with suspected lung injury who needs intubation for acute respiratory failure may be of benefit. However, certain situations may preclude their use [36].

### Importance of This Study

As outlined above, temporal changes in airway and sedation management in recent years have occurred with mixed study results and the importance of short-term outcomes (ie, postintubation hypotension, hypoxemia, and difficult airway) on patient morbidity and mortality has been demonstrated. Therefore, characterization of current intubation practice among the critically ill is warranted. With this characterization, scoring systems may be developed that aid the clinician in providing optimal outcomes for patients. This information in turn may allow the clinician to provide a tailored plan, in terms of both airway and hemodynamic management of the critically ill who are in need of intubation.

### Approach

In order to examine the current endotracheal intubation practice among the critically ill, a multicenter, observational, prospective study of adult, critically ill patients was conducted from July 2015 to January 2017 involving 20 ICUs.

### Study Objectives

Our first objective is to derive and validate a prediction model for airway difficulty among the critically ill as defined by three or more attempts at laryngoscopy and/or the need for another operator [37].

Our second objective is to derive and validate a prediction model for hemodynamic compromise (ie, postintubation hypotension, defined as a decrease at any point in mean arterial pressure of <65 mmHg; systolic blood pressure of <80 mmHg and/or a decrease in systolic blood pressure of 40% from previous; or the introduction, or increase in infusion rate, of any vasoactive agent during the 30-minute window following endotracheal intubation) [38,39].

Our third objective is to derive and validate a predication model for hypoxemia defined as a decrease in SpO_2_ of <88% during the procedure.

## Methods

### Study Approval and Trial Registration

The Institutional Review Board at the Mayo Clinic in Rochester, Minnesota, approved this study protocol (Institutional Review Board #15-002328). This study was under waiver of informed consent and authorization given the observational nature of the study with the use of deidentified data. The trial was registered at ClinicalTrials.gov (#NCT02508948).

### Design and Setting

This is a multicenter, observational, prospective study of adult critically ill patients admitted to both medical and surgical ICUs at the listed participating sites within the United States (see Multimedia Appendix 1), who meet the criteria designated below for which endotracheal intubation was needed.

### Inclusion and Exclusion Criteria

Inclusion and exclusion criteria for the study are listed in Textbox 1.

### Study Enrollment Procedures

All adult endotracheal intubations across all ICUs were eligible for this study. Given that this procedure is an unpredictable event, the patients were not able to consent, nor was a health care power of attorney readily available. The sites were to initiate immediate data collection; therefore, obtaining informed consent was impractical. In addition, the observational study design did not impact the procedures performed, devices used, or medications given to patients.

### Study Protocol

A steering committee oversaw the administration of the protocol and was comprised of both anesthesia and pulmonary critical care physicians. A data collection form was created that focused on two periprocedural aspects of the intubation process, including airway and hemodynamic management, and was used at all participating sites (see Multimedia Appendix 2). Regarding airway management, rapid sequence intubation was defined a priori according to Sellick [40]. Although the participating sites obtained formal training in the use of the data collection form prior to study initiation, online content in the form of a web link was established to answer frequently asked questions, as well as to establish a forum among the investigators with all questions related to the study discussed (see Multimedia Appendix 3). Moreover, monthly HEModynamic and AIRway (HEMAIR) investigator meetings were conducted to further provide a platform for questions and discuss future collaborations, with a newsletter sent afterward to participating sites (see Multimedia Appendix 3). The data collection form was uploaded into RedCap during data entry. Data were obtained by the proceduralist or site study coordinator and verified by the primary investigative team. The sampling method utilized in this study was convenience sampling.

### Data Management

Each clinical site was responsible for patient enrollment and data collection. Each site also provided a research investigator who was responsible for capturing and entering the study data into the study database during the collection time period. The study database was housed and managed at the Mayo Clinic in Rochester, Minnesota, including running periodic, basic data-quality-monitoring queries. Data collection on outcome measures was done weekly by trained study coordinators at each site.

### Statistical Analysis

For descriptive summaries, continuous measurements will be represented as mean (SD) for parametric distributions and median (interquartile range, IQR) for nonparametric distributions. Dichotomous variables will be represented as counts and percentages. For descriptive studies, all procedures will be included for patients who require endotracheal intubation more than once during the same ICU stay. For hypothesis testing, we will consider two-tailed tests of *P*<.05 to be statistically significant and will report point estimates and 95% confidence intervals. The first endotracheal intubation in the ICU will be used in analyses to assess for associations between predictors and an adverse outcome. Model building will be performed using lasso regression with 10-fold cross-validation. In all cases, distributional assumptions will be assessed, with appropriate transformations used as necessary. SAS version 9.4 (SAS Institute Inc) and R statistical software version 3.4.1 (R Foundation for Statistical Computing) will be used for all analyses.

**Inclusion and exclusion criteria.**

Inclusion criteria:

- Adult patients (≥18 years of age)
- Admission to medical or surgical intensive care unit
- Endotracheal intubation performed between July 2015 and January 2017

Exclusion criteria:

- Pediatric patients (<18 years of age)
- Endotracheal intubations occurring in nonintensive care unit locations

### Sample Size

We based our sample size on the occurrence of intubation complications. Since we are most concerned with the occurrence of airway-related complications (ie, difficult intubation), and hemodynamic complications (ie, hypotension), our sample size is powered for the occurrence of these two complications. Difficult intubation and hypotension were defined in our study with an expected incidence of 12% and 11%, respectively, during the peri-intubation period [37,41]. Thus, we determined that an effective sample of 804 was sufficient to provide statistical power to detect an incidence of 12% with precision and margin of error of 1%. However, we included over 1000 patients from all sites to answer any subsequent secondary and tertiary hypotheses.

## Results

The study is currently undergoing data analysis. Results are expected in November 2018 with publication to follow thereafter. The study protocol has not yet undergone peer review by a funding body.

## Discussion

The HEMAIR study did not alter the care that patients received. Additionally, sharing deidentified data protected the privacy of the patients. With these procedures and requirements in place, the physical rights and welfare of patients were not adversely affected by study participation or by the waiver of consent and authorization. This multicenter, prospective trial will be among the first to include a large, diverse patient population from across the United States with a large sample size. The potential benefits would include deriving and validating prediction models for immediate severe complications regarding airway and hemodynamic management surrounding intubations among the critically ill. With this information, it is our hope that clinicians will have a tool to predict which patients will become unstable during this procedure so they may adjust treatment plans, allowing for improved quality of care delivered during this procedure. This prospective observational trial is even more important, as postintubation hypotension and hemodynamic derangement is noted by some to occur at a fairly high rate, possibly leading to increased risk of mortality [42].

## Appendix

Multimedia Appendix 1 HEModynamic and AIRway (HEMAIR) site investigators.

Multimedia Appendix 2 HEModynamic and AIRway (HEMAIR) data collection form.

Multimedia Appendix 3 HEModynamic and AIRway (HEMAIR) newsletter.

## Abbreviations

HEMAIR: HEModynamic and AIRway
ICU: intensive care unit
IQR: interquartile range
