# Supplementary material for: Endotracheal Intubation Among the Critically Ill: Protocol for a Multicenter, Observational, Prospective Study
Source: JMIR Res Protoc. 2018 Dec 7;7(12):e11101. doi: 10.2196/11101 (PMC6303735; doi:10.2196/11101)
Supplement: Multimedia Appendix 1 [file resprot_v7i12e11101_app1.pdf]

No	HHS	Participating ICUs	City	State	Principal Investigator
1	5	Aurora Healthcare	Milwaukee	Wisconsin	Ernesto Brauer, MD
2	1	Berkshire Medical Center	Pittsfield	Massachusetts	Cynthia Callahan, MD
3	1	Bridgeport Hospital/Yale	Bridgeport	Connecticut	David Kaufman, MD
4	5	Cleveland Clinic	Cleveland	Ohio	Ashish Khanna, MD
5	7	Creighton University	Omaha	Nebraska	Lee E. Morrow, MD, MSc
6	6	Corpus Christi Medical Center	Corpus Christi	Texas	Salim Surani, MD
7	5	Detroit Medical Center	Detroit	Michigan	Sarah Lee, MD
8	8	Essentia Health, Fargo	Fargo	North Dakota	Jaise Poulose, MD
9	3	Geisinger Health System	Danville	Pennsylvania	Uchenna Ofoma, MD
10	3	Johns Hopkins-HCGH	Columbia	Maryland	William Checkley, MD
11	5	Marshfield Clinic	Marshfield	Wisconsin	Jayanth Vedre, MD
12	4	Mayo Clinic, Jacksonville	Jacksonville	Florida	Pablo Moreno, MD
13	5	Mayo Clinic, Rochester	Rochester	Minnesota	Nathan Smischney, MD, MSc
14	9	Mayo Clinic, Scottsdale	Scottsdale	Arizona	Ayen Sen, MD
15	9	Memorial Medical Center	Modesto	California	Rudy Tedja, MD
16	7	Mercy Hospital	Saint Louis	Missouri	Chakradhar Venkata, MD
17	4	University of Kentucky	Lexington	Kentucky	Peter Morris, MD
18	4	University of North Carolina	Chapel Hill	North Carolina	Thomas Bice, MD
19	6	University of Oklahoma Health Sciences Center	Oklahoma City	Oklahoma	Gozde Demiralp, MD
20	9	University of South California	Los Angeles	California	Santhi Iyer Kumar, MD
