# Supplementary material for: Endotracheal Intubation Among the Critically Ill: Protocol for a Multicenter, Observational, Prospective Study
Source: JMIR Res Protoc. 2018 Dec 7;7(12):e11101. doi: 10.2196/11101 (PMC6303735; doi:10.2196/11101)
Supplement: Multimedia Appendix 2 [file resprot_v7i12e11101_app2.pdf]

Demographics & Co-morbidities

Demographics

Center ID ________________________________

Patient Study ID ________________________________

Hospital admission date-time ________________________________

ICU admission date-time ________________________________

Intubation date-time ________________________________

Year of Birth (Only include, if >18 years) ________________________________
(YYYY)

Gender
☐ Male
☐ Female

Weight ________________________________
(kg.)

Height ________________________________
(cm.)

History of previous difficult tracheal intubation
☐ Yes
☐ No
☐ Not known

Explain_previous difficult tracheal intubation ________________________________

Co-morbidities--from past medical history, History of:

	Yes	No
Congestive Heart Failure	☐	☐
Ischemic Heart (Coronary Artery) disease	☐	☐
COPD	☐	☐
ESRD	☐	☐
Cirrhosis	☐	☐
Diabetes Mellitus	☐	☐

Before Intubation, if any of these were present:

	Yes	No
Acute Kidney Injury=/>Stage 1	☐	☐
Dialysis or CRRT	☐	☐
Mechanical circulatory support	☐	☐

Sepsis	☐	☐
Hypovolemic Shock	☐	☐

Intubation Process

Laboratory results (close to and within 6 hours of tracheal intubation)

Hemoglobin Level ______________________
(In gram per deciliter)

Lactate Level ______________________
(mmol/L (if NA in 6 hours, enter closest value in last 24 hours))

Lactate and Hb date-time ______________________

Emergency of tracheal Intubation

Emergency of tracheal Intubation
- ☐ Emergency (ETI required without any delay)
- ☐ Urgency (ETI required in < 1 hour)
- ☐ Elective (non-emergent/procedural related)

Reasons for tracheal intubation (check all that apply)

	Yes	No
Airway Protection	☐	☐
Acute respiratory failure: Dyspnea and/or Sao2< 90%	☐	☐
Neurologic: Stroke or altered mental status	☐	☐
Cardiac arrest	☐	☐
Shock : MAP < 65 mm Hg	☐	☐
Procedural-related (e.g. intubation for upper endoscopy or bronchoscopy)	☐	☐

Tracheal intubation process

Did the patient suffer cardiac arrest requiring CPR during intubation ?
- ☐ yes
- ☐ no

Calculated APACHE II (first 24 hours) score
- ☐ Yes
- ☐ No

APACHE II Score 24 hours prior to intubation ______________________

APACHE II age
- ☐ < or = 44
- ☐ 45-54
- ☐ 55-64
- ☐ 65-74
- ☐ = or >74

((Years))

APACHE II Hematocrit

- ☐ < 20%
- ☐ 20-29.9%
- ☐ 30-45.9%
- ☐ 46-49.9%
- ☐ 50-59.9%
- ☐ 60%

APACHE II WBC

- ☐ < 1000
- ☐ 1000-2999
- ☐ 3000-14999
- ☐ 15000-19999
- ☐ 20000-39999
- ☐ = or >40000

(per micro liter)

APACHE II Rectal Temperature

- ☐ > or = 41
- ☐ 39-40.9
- ☐ 38.5-38.9
- ☐ 36-38.4
- ☐ 34-35.9
- ☐ 32-33.9
- ☐ 30-31.9
- ☐ < or = 29.9

(Degrees celsius)

APACHE II Mean Arterial Pressure

- ☐ < or = 49
- ☐ 50-69
- ☐ 70-109
- ☐ 110-129
- ☐ 130-159
- ☐ = or >160

(MAP=[(2 x diastolic) + systolic] / 3))

APACHE II Heart rate

- ☐ < or = 30
- ☐ 40-54
- ☐ 55-69
- ☐ 70-109
- ☐ 110-139
- ☐ 140-179
- ☐ = or >180

(beats per minute)

APACHE II Respiratory rate

- ☐ < or =5
- ☐ 6-9
- ☐ 10-11
- ☐ 12-24
- ☐ 25-34
- ☐ 35-49
- ☐ > or =50

(breaths per minute)

APACHE II Serum sodium

- ☐ > or = 180
- ☐ 160-179
- ☐ 155-159
- ☐ 150-154
- ☐ 130-149
- ☐ 120-129
- ☐ 111-119
- ☐ < or = 110

(mEq/L)

APACHE II FiO2

____

((FiO2 (fraction of inspired oxygen) FiO2 on room air = 0.21))

APACHE II PaO2

____

((mm Hg, PaO2 (partial pressure of arterial O2) from ABG))

APACHE II PaCo2

____
((mm Hg, PaO2 (partial pressure of arterial O2) from ABG))

APACHE II Arterial pH

____
((xx.xx))

APACHE II HCo3

____
((xx.x))

APACHE II Serum creatinine

- ☐ > or + 3.5 and NOT acute renal failure
- ☐ 2.0 - 3.4 and NOT acute renal failure
- ☐ 1.5 - 1.9 and NOT acute renal failure
- ☐ 0.6 - 1.4 and NOT acute renal failure
- ☐ < 0.6 and NOT acute renal failure
- ☐ > or = 3.5 and acute renal failure
- ☐ 2.0 - 3.4 and acute renal failure
- ☐ 1.5 - 1.9 and acute renal failure
- ☐ 0.6 - 1.4 and acute renal failure
- ☐ < 0.6 and acute renal failure

((mg per dl))

APACHE II History of severe organ insufficiency (heart, liver, kidney, other) or immunocompromised

- ☐ NO
- ☐ YES and None - operative patient
- ☐ YES and Emergency post-operative patient
- ☐ YES and Elective surgery post-operative patient

APACHE II GCS EYE OPENING

- ☐ Spontaneous
- ☐ To command
- ☐ To pain
- ☐ No response

((Glasgow coma scoring -Eye))

APACHE II GCS Verbal

- ☐ Oriented
- ☐ Confused
- ☐ Inappropriate
- ☐ Incomprehensible
- ☐ No Response

((Glasgow coma Scoring - Verbal))

APACHE II GCS Motor

- ☐ Obeys Commands
- ☐ Localizes pain
- ☐ Withdraws to pain
- ☐ Flexion to pain
- ☐ Extension to pain
- ☐ No Response

((Glasgow coma Scoring - Motor))

Non invasive Ventilation 60 mins Prior

- ☐ None
- ☐ CPAP
- ☐ BiPAP

Use of any preoxygenation

- ☐ Nasal cannula
- ☐ Simple facemask
- ☐ Non re-breather facemask
- ☐ Bag mask ventilation
- ☐ Optiflow

Mask Ventilation

- ☐ Easy
- ☐ Oral/Nasal airway needed
- ☐ Difficult (i.e. two handed)
- ☐ Impossible (no air movement despite any technique)

What provider level performed the primary/final airway management technique

- ☐ Consultant/Attending
- ☐ Fellow
- ☐ Resident
- ☐ Respiratory Therapist
- ☐ Medical student
- ☐ Nurse practitioner/ Physician assistant
- ☐ Certified registered nurse anesthetist
- ☐ EMT

Primary/final airway management technique

- ☐ Mask
- ☐ Macintosh direct laryngoscopy
- ☐ Miller direct laryngoscopy
- ☐ Video laryngoscopy
- ☐ Fiber optic broncoscopy
- ☐ Cricothyrotomy
- ☐ Tracheostomy
- ☐ Retrograde
- ☐ Cook Arndt exchange catheter
- ☐ Blind Nasal
- ☐ Trans tracheal jet ventilation
- ☐ LMA
- ☐ Intubating LMA
- ☐ King airway
- ☐ combitube
- ☐ Gum elastic bougie

Other techniques beside final airway management technique

- ☐ None
- ☐ Mask
- ☐ Macintosh direct laryngoscopy
- ☐ Miller direct laryngoscopy
- ☐ Video laryngoscopy
- ☐ Fiber optic broncoscopy
- ☐ Cricothyrotomy
- ☐ Tracheostomy
- ☐ Retrograde
- ☐ Cook Arndt exchange catheter
- ☐ Blind Nasal
- ☐ Trans tracheal jet ventilation
- ☐ LMA
- ☐ Intubating LMA
- ☐ King airway
- ☐ combitube
- ☐ Gum elastic bougie

Number of intubation attempts(Mask)

____
(Attempt = insertion of device (laryngoscope or video laryngoscope) into oral cavity. Removal from oral cavity then re-insertion would constitute another attempt)

Number of intubation attempts(Machintosh direct laryngoscopy)

____
(Attempt = insertion of device (laryngoscope or video laryngoscope) into oral cavity. Removal from oral cavity then re-insertion would constitute another attempt)

Number of intubation attempts(Miller direct laryngoscopy)

____
(Attempt = insertion of device (laryngoscope or video laryngoscope) into oral cavity. Removal from oral cavity then re-insertion would constitute another attempt)

Number of intubation attempts(Video laryngoscopy)

____
(Attempt = insertion of device (laryngoscope or video laryngoscope) into oral cavity. Removal from oral cavity then re-insertion would constitute another attempt)

Number of intubation attempts(Fiber optic broncoscopy) ________________________________
(Attempt = insertion of device (laryngoscope or video laryngoscope) into oral cavity. Removal from oral cavity then re-insertion would constitute another attempt)

Number of intubation attempts(Cricothyrotomy) ________________________________
(Attempt = insertion of device (laryngoscope or video laryngoscope) into oral cavity. Removal from oral cavity then re-insertion would constitute another attempt)

Number of intubation attempts(Retrograde) ________________________________
(Attempt = insertion of device (laryngoscope or video laryngoscope) into oral cavity. Removal from oral cavity then re-insertion would constitute another attempt)

Number of intubation attempts(Cook Arndt exchange catheter) ________________________________
(Attempt = insertion of device (laryngoscope or video laryngoscope) into oral cavity. Removal from oral cavity then re-insertion would constitute another attempt)

Number of intubation attempts(Blind Nasal) ________________________________
(Attempt = insertion of device (laryngoscope or video laryngoscope) into oral cavity. Removal from oral cavity then re-insertion would constitute another attempt)

Number of intubation attempts(LMA) ________________________________
(Attempt = insertion of device (laryngoscope or video laryngoscope) into oral cavity. Removal from oral cavity then re-insertion would constitute another attempt)

Number of intubation attempts(Intubating LMA) ________________________________
(Attempt = insertion of device (laryngoscope or video laryngoscope) into oral cavity. Removal from oral cavity then re-insertion would constitute another attempt)

Number of intubation attempts(King airway) ________________________________
(Attempt = insertion of device (laryngoscope or video laryngoscope) into oral cavity. Removal from oral cavity then re-insertion would constitute another attempt)

Number of intubation attempts(combitube) ________________________________
(Attempt = insertion of device (laryngoscope or video laryngoscope) into oral cavity. Removal from oral cavity then re-insertion would constitute another attempt)

Number of intubation attempts(Gum elastic bougie) ________________________________
(Attempt = insertion of device (laryngoscope or video laryngoscope) into oral cavity. Removal from oral cavity then re-insertion would constitute another attempt)

Route of tracheal Intubation on final attempt
- ☐ Nasal
- ☐ Oral
- ☐ Surgical

Confirmation of tracheal tube placement (check all that apply)
- ☐ Auscultation/ Chest rise
- ☐ ETC02
- ☐ CXR
- ☐ Bronchoscopy

Immediate complication of tracheal intubation (check all that apply)

- ☐ None
- ☐ Esophageal intubation(recognized immediately)
- ☐ Esophageal intubation(delayed recognized)
- ☐ Dental trauma
- ☐ Vomiting
- ☐ Oral or Laryngeal trauma
- ☐ Medication error
- ☐ Laryngospasm
- ☐ Nasal bleeding
- ☐ Malignant hyperthermia
- ☐ Hypotension
- ☐ Arrhythmia
- ☐ Mainstem intubation
- ☐ Pneumothorax
- ☐ Cardiac arrest
- ☐ Cardiovascular collapse
- ☐ Hypoxemia
- ☐ Aspiration
- ☐ Other

(within 30 minutes after tracheal intubation)

Other immediate complication of tracheal intubation ______________________________

If direct or video laryngoscopy, then complete the following questions

Glottis exposure (Cormack and Lehane grade)

- ☐ Grade I
- ☐ Grade II
- ☐ Grade III
- ☐ Grade IV

Lifting force required during laryngoscopy

- ☐ Normal
- ☐ Increased

External Laryngeal Pressure

- ☐ Not applied
- ☐ Applied

(Cricoid pressure/ Sellick maneuver)

Position of vocal cords at tracheal intubation

- ☐ Abduction
- ☐ Adduction
- ☐ Not observed

Events within 60 minutes post intubation

Set Tidal Volume ______________________________
(ml)

Positive end-expiratory pressure ______________________________
(mm of Hg)

comments for intubation process section ______________________________

Vital signs, Intubation Medications and Fluids Infused

Vital parameter 60 minutes before Intubation (if not at 60 mins, enter value closest to 60 mins)

SBP ______________________ (mm of Hg)

DBP ______________________ (mm of Hg)

Heart Rate ______________________ (per minute)

Respiratory rate ______________________ (per minute)

Pulse oxymetry ______________________ (SpO2)

Temperature ______________________ (fahrenheit-F)

Vital parameter 30 minutes before Intubation (if not at 30 mins, enter value closest to 30 mins)

SBP ______________________ (mm of Hg)

DBP ______________________ (mm of Hg)

Heart Rate ______________________ (per minute)

Respiratory rate ______________________ (per minute)

Pulse oxymetry ______________________ (SpO2)

Vital parameter right before Intubation (within 15 mins prior)

Mention time for vital parameter right before Intubation (within 15 mins prior) ______________________

SBP ______________________ (mm of Hg)

DBP ______________________ (mm of Hg)

Heart Rate ______________________ (per minute)

Respiratory rate ______________________ (per minute)

Pulse oxymetry ______________________ (SpO2)

Vital parameters right after Intubation (within 15 mins)

Mention time for vital parameter right after Intubation (within 15 mins post) ________________

SBP ________________ (mm of Hg)

DBP ________________ (mm of Hg)

Heart Rate ________________ (per minute)

Respiratory rate ________________ (per minute)

Pulse oxymetry ________________ (SpO2)

Vital parameters 30 mins after Intubation (if not at 30 mins, enter value closest to 30 mins)

SBP ________________ (mm of Hg)

DBP ________________ (mm of Hg)

Heart Rate ________________ (per minute)

Respiratory rate ________________ (per minute)

Pulse oxymetry ________________ (SpO2)

Vital parameters 60 mins after Intubation (if not at 60 mins, enter value closest to 60 mins)

SBP ________________ (mm of Hg)

DBP ________________ (mm of Hg)

Heart Rate ________________ (per minute)

Respiratory rate ________________ (per minute)

Pulse oxymetry ________________ (SpO2)

Temperature ________________ (fahrenheit-F)

Any sedative/hypnotic medications within 60 minutes around tracheal intubation

Any sedative/hypnotic medications within 60 minutes pre - intubation

- ☐ None
- ☐ Lorazepam
- ☐ Midazolam
- ☐ Propofol
- ☐ Ketamine
- ☐ Dexmedetomidine
- ☐ Fentanyl
- ☐ Morphine
- ☐ Hydromorphone
- ☐ Other

Other_sedative/hypnotic medications within 60 minutes pre-intubation ________________________________

Any sedative/hypnotic medications within 60 minutes post - intubation

- ☐ None
- ☐ Lorazepam
- ☐ Midazolam
- ☐ Propofol
- ☐ Ketamine
- ☐ Dexmedetomidine
- ☐ Fentanyl
- ☐ Morphine
- ☐ Hydromorphone
- ☐ Other

Other_sedative/hypnotic medications within 60 minutes post - intubation ________________________________

Fluid bolus within 60 minutes around intubation

Fluid Bolus (60 minutes pre intubation)

- ☐ Yes
- ☐ No

(Defined as >500ml of crystalloid/colloid)

Fluid Bolus (if yes mention quantity) ________________________________

Fluid Bolus (60 minutes post intubation)

- ☐ Yes
- ☐ No

(Defined as >500ml of crystalloid/colloid)

Fluid Bolus (if yes mention quantity) ________________________________

Cardiovascular medications 24 hours Pre-Intubation

Cardiovascular medications 24 hours Pre-Intubation

- ☐ None
- ☐ Diuretics
- ☐ Alpha blockers
- ☐ Clonidine
- ☐ Ace Inhibitors/ARB
- ☐ Midodrine
- ☐ Nitrates
- ☐ Calcium channels blockers
- ☐ Anti arrhythmics
- ☐ Others

Others Cardiovascular medication 24 hours Pre-Intubation ________________________________

Intubation Medication

	Yes	No
Ketamine	☐	☐
Propofol	☐	☐
Etomidate	☐	☐
Ketamine / propofol admixture	☐	☐
Fentanyl	☐	☐
Midazolam	☐	☐
Succinylcholine	☐	☐
Rocuronium	☐	☐
Vecuronium	☐	☐
Atracurium	☐	☐
Cisatracurium	☐	☐
Morphine	☐	☐
Hydromorphone	☐	☐
Lorazepam	☐	☐
Lidocaine	☐	☐
Sufentanil	☐	☐
Thiopental	☐	☐
Other Intubation Medication	☐	☐

Ketamine ____________________
(Total dose in milligrams)

Propofol ____________________
(Total dose in milligrams)

Etomidate ____________________
(Total dose in milligrams)

Ketamine /Propofol Admixture ____________________
(Total dose of each in milligrams. For Ex: 0.5+0.5)

Fentanyl ____________________
(Total dose in micrograms)

Midazolam ____________________
(Total dose in milligrams)

Succinylcholine ____________________
(Total dose in milligrams)

Rocuronium ____________________
(Total dose in milligrams)

Vecuronium ____________________
(Total dose in milligrams)

Atracurium ____________________
(Total dose in milligrams)

Cisatracurium ____________________
(Total dose in milligrams)

Morphine ______________________ (Total dose in milligrams)

Hydromorphone ______________________ (Total dose in milligrams)

Lorazepam ______________________ (Total dose in milligrams)

Lidocaine ______________________ (Total dose in milligrams)

Sufentanil ______________________ (Total dose in micrograms)

Thiopental ______________________ (Total dose in milligrams)

Other Intubation Medication ______________________ (Total dose in milligrams)

Use of Anticholinergics (or) B-blockers during Intubation

	Yes	No
Atropine	☐	☐
Glycopyrrolate	☐	☐
Esmolol	☐	☐
Metoprolol	☐	☐
Use of other Anticholinergics (or) B-blockers during Intubation	☐	☐

Atropine ______________________ (Total dose in milligrams)

Glycopyrrolate ______________________ (Total dose in milligrams)

Esmolol ______________________ (Total dose in milligrams)

Metoprolol ______________________ (Total dose in milligrams)

Use of other Anticholinergics (or) B-blockers during Intubation ______________________ (Total dose in milligrams)

Vasopressor use 60 mins before tracheal intubation --> look at dose in 15 minute intervals and then multiply dose in that 15 minute interval by 15 mins and then add the four 15 min doses together to get the total 60 min dose

	Yes	No
Calcium (Used specifically for hemodynamics)	☐	☐
Dopamine	☐	☐
Epinephrine	☐	☐
Norepinephrine	☐	☐
Vasopressin	☐	☐
Phenylephrine	☐	☐
Dobutamine	☐	☐
Milrinone	☐	☐
Ephedrine	☐	☐
Other vasopressor used	☐	☐

Calcium ______________________ (Used specifically for hemodynamics, in grams)

Dopamine ______________________ (Total dose in micrograms)

Epinephrine ______________________ (Total dose in micrograms)

Norepinephrine ______________________ (Total dose in micrograms)

Vasopressin ______________________ (Total dose in units)

Phenylephrine ______________________ (Total dose in micrograms)

Dobutamine ______________________ (Total dose in micrograms)

Milrinone ______________________ (Total dose in micrograms)

Ephedrine ______________________ (Total dose in milligrams)

Other Vasopressors used ______________________ (Total dose in milligrams)

Vasopressor use 60 mins after tracheal intubation --> look at dose in 15 minute intervals and then multiply dose in that 15 minute interval by 15 mins and then add the four 15 min doses together to get the total 60 min dose

	Yes	No
Calcium (Used specifically for hemodynamics)	☐	☐
Dopamine	☐	☐
Epinephrine	☐	☐
Norepinephrine	☐	☐
Vasopressin	☐	☐
Phenylephrine	☐	☐
Dobutamine	☐	☐
Milrinone	☐	☐
Ephedrine	☐	☐
Other Vasopressor used	☐	☐

Calcium ______________________ (Used specifically for hemodynamics, in grams)

Dopamine ______________________ (Total dose in micrograms)

Epinephrine ______________________ (Total dose in micrograms)

Norepinephrine ______________________ (Total dose in micrograms)

Vasopressin ______________________ (Total dose in units)

Phenylephrine ______________________ (Total dose in micrograms)

Dobutamine ______________________ (Total dose in micrograms)

Milrinone ______________________ (Total dose in micrograms)

Ephedrine ______________________ (Total dose in milligrams)

Other Vasopressors used ______________________ (Total dose in milligrams)

Volume 24 hours prior to tracheal intubation

Total crystalloid volume (ml) ______________________

Total colloid volume excluding blood products (ml) ______________________

Total packed RBCs (units) ______________________

Total fresh frozen plasma (FFP) (units) ______________________

Total platelet volume (units) ________________________________

Total cryoprecipitate volume (units) ________________________________

Total urine output (ml) ________________________________

Volume 24 hours after tracheal intubation

Total crystalloid volume (ml) ________________________________

Total colloid volume excluding blood products (ml) ________________________________

Total packed RBCs (units) ________________________________

Total fresh frozen plasma (FFP) (units) ________________________________

Total platelet volume (units) ________________________________

Total cryoprecipitate volume (units) ________________________________

Total urine output (ml) ________________________________

Comments for Vital signs, Intubation medications and Fluids infused section ________________________________

Outcome

Syndromes in ICU after tracheal intubation (24 hours following)

	Yes	No
Acute Kindey Injury-Stage1 or higher	☐	☐
Dialysis or Continuous Renal Replacement Therapy	☐	☐
Sepsis	☐	☐
Hypovolemic Shock	☐	☐

Mortality and Follow-up

Number of days on MV during ICU Stay ________________________________
(Mechanical Ventilation)

Tracheostomy required during ICU stay
☐ Yes
☐ No

Final Discharge Diagnosis- Primary ________________________________
(If required, explain in comments below)

Final Discharge Diagnosis- Secondary
(If more than one, write all - separated by comma)

ICU discharge date ________________________________

ICU discharge status: Dead
☐ Yes
☐ No

Hospital discharge date ________________________________

Hospital discharge status: Dead
☐ Yes
☐ No

Patient was discharged to
☐ Home
☐ Other hospital
☐ Nursing home
☐ Rehabilitation center
☐ Other

Comments for outcome section ________________________________
