# Supplementary material for: Endotracheal Intubation Among the Critically Ill: Protocol for a Multicenter, Observational, Prospective Study
Source: JMIR Res Protoc. 2018 Dec 7;7(12):e11101. doi: 10.2196/11101 (PMC6303735; doi:10.2196/11101)
Supplement: Multimedia Appendix 3 [file resprot_v7i12e11101_app3.pdf]

HEMAIR STUDY NEWSLETTER

Cumulative Patient Enrollment --- as of November 21, 2016

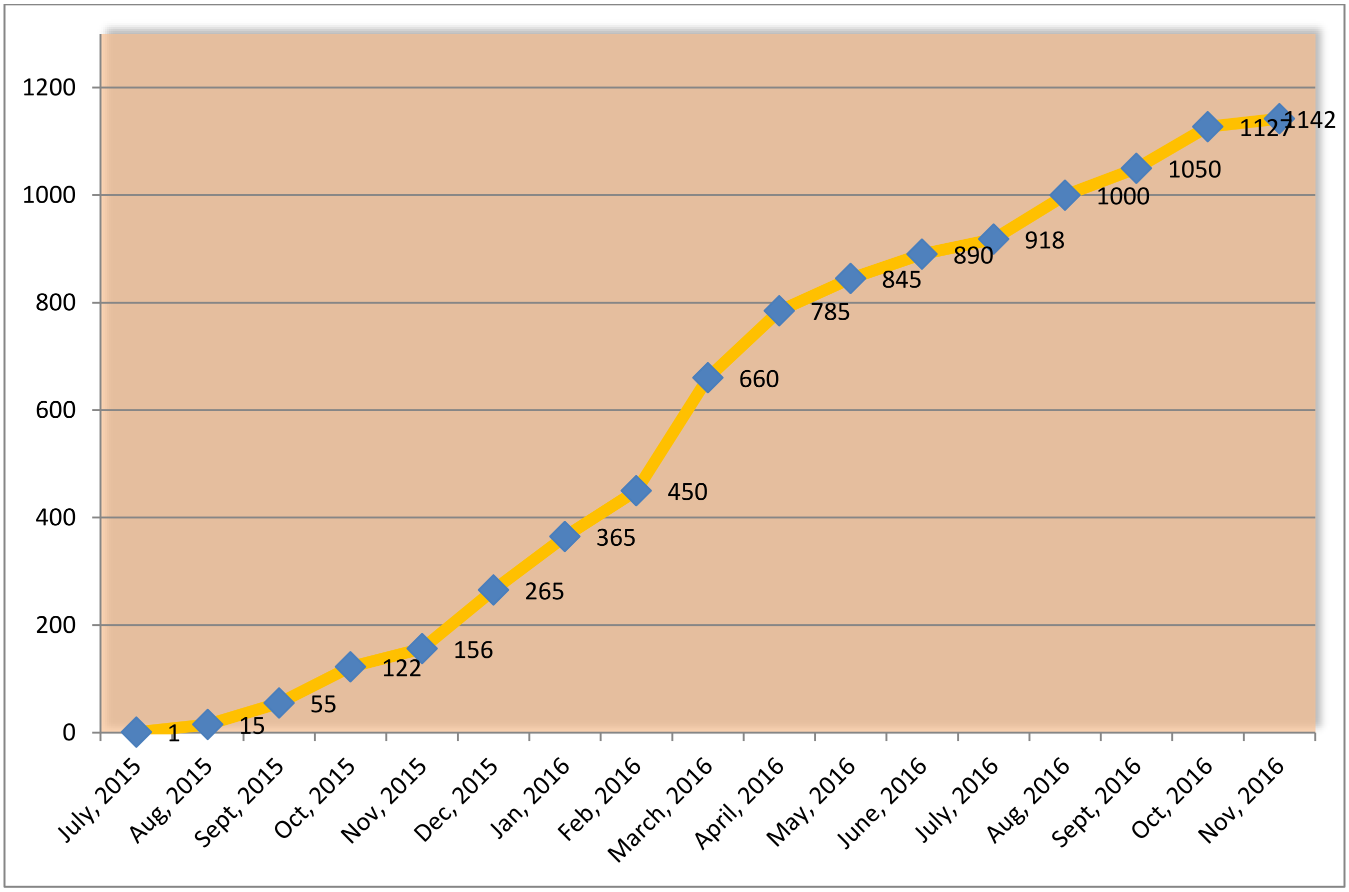

Total 1142 enrollments

No	Participating ICUs	City	State	IRB approved	Enrolled Patients
1	Akron General Medical Center	Akron	Ohio		
2	Aurora Healthcare	Milwaukee	Wisconsin	yes	130
3	Berkshire Medical Center	Pittsfield	Massachusetts	yes	61
4	Beth Israel Deaconess Medical Center	Boston	Massachusetts		
5	Bridgeport Hospital/Yale	Bridgeport	Connecticut	yes	61
6	Cleveland Clinic	Cleveland	Ohio	yes	111

**7**	Creighton University	Omaha	Nebraska	yes	100
**8**	Corpus Christi Medical Center, HCA Campus	Corpus Christi	Texas	yes	5
**9**	D. P. Phillips Hospital	Orlando	Florida		
**10**	Dartmouth-Hitchcock Medical Center	Lebanon	New Hampshire		
**11**	Detroit Medical Center	Detroit	Michigan	yes	6
**12**	Essentia Health, Fargo	Fargo	North Dakota	yes	5
**13**	Geisinger Health System	Danville	Pennsylvania	yes	97
**14**	Johns Hopkins-HCGH	Columbia	Maryland	yes	
**15**	Kaiser Permanente	Redwood City	California		
**16**	Keck Hospital of USC	Los Angeles	California		
**17**	Marshfield Clinic	Marshfield	Wisconsin	yes	4
**18**	Mayo Clinic, Jacksonville	Jacksonville	Florida	yes	93
**19**	Mayo Clinic, Rochester	Rochester	Minnesota	yes	166
**20**	Mayo Clinic, Scottsdale	Scottsdale	Arizona	yes	65
**21**	Memorial Hospital of Rhode Island	Pawtucket	Rhode Island		
**22**	Memorial Medical Center	Modesto	California	yes	50
**23**	Mercy Hospital	Saint Louis	Missouri	yes	57
**24**	University of Arizona	Tucson	Arizona		
**25**	University of Kentucky	Lexington	Kentucky	yes	79
**26**	University of Michigan	Ann Arbor	Michigan		
**27**	University of North Carolina	Chapel Hill	North Carolina	yes	1
**28**	University of Oklahoma Health Sciences Center	Oklahoma City	Oklahoma	yes	46
**29**	University of South California	Los Angeles	California	yes	5

Latest Frequently Asked Questions (FAQs) –

1. What if the patient has already been intubated in OR and later admitted into ICU got extubated and then re-intubated for genuine reason do we need to enroll these patients into the study?

A. Yes! We would enroll these patients into the study. If, the patient has been extubated and then re-intubated later for a genuine reason like respiratory failure. If, the patient has pulled out his ET-tube then he/she can be excluded from the study as it is not the first time genuine ICU intubation.

2. Tell me more about the data management ownership and secondary ancillary studies?

A. The participating centers will own the data that they have collected at their center. Members of the HEMAIR group will be able to access the complete data for ancillary research projects provided the proposal has been reviewed and deemed satisfactory by the HEMAIR Steering committee.

3. Non-invasive Cardiac output Monitoring in ICU (INCOM) Study.

Mayo Clinic Rochester and Bridgeport Hospital /Yale will start this study once the IRBe is approved using NICOM device. Both centers will enroll 40 patients or the study.

4. Data Quality and Cleaning.

Our team at Rochester has started data quality check and they are sending data quality report each month to each center and they will continue to do so until the whole data is cleaned or we achieve require numbers of enrollments.

FAQs will be updated to all the participating centers by emails and can be found at http://www.hemairregistry.org

Next executive call details :

Monday, Dec 19th, 2016, Time: 03:00 – 03:45 PM CDT
This is a recurring meeting every third Monday of each month 03:00 – 03:45 PM CDT.
Join from PC, Mac, iOS or Android: https://zoom.us/j/865220624
Please come online few minutes earlier to set up zoom for the first time!
Or join by phone:
+1 415 762 9988 (US Toll) or +1 646 568 7788 (US Toll)
Meeting ID: 865 220 624
International numbers available: https://zoom.us/zoomconference

Study Websites : www.hemairregistry.org

Study Contacts :

Protocol, IRB and Ancillary Studies: Smischny.Nathan@mayo.edu; Kashyap.Rahul@mayo.edu;
Training and Implementation: Kashyap.Rahul@mayo.edu; Kumar.Mukesh@mayo.edu; Seisa.Mohmed@mayo.edu

Upcoming HEMAIR Investigator Meetings

At SCCM 2017: Jan 22nd, 2017 at 4:30 pm
Location: Hawaii Convention Center, Honolulu, Hawaii, USA

At ATS 2017: May 22nd, 2017 at 4:30 pm
Location: Washington DC.
